# Identification of Newly Synthesized Proteins by *Echinococcus granulosus* Protoscoleces upon Induction of Strobilation

**DOI:** 10.1371/journal.pntd.0004085

**Published:** 2015-09-22

**Authors:** João Antonio Debarba, Karina Mariante Monteiro, Hercules Moura, John R. Barr, Henrique Bunselmeyer Ferreira, Arnaldo Zaha

**Affiliations:** 1 Programa de Pós-Graduação em Biologia Celular e Molecular, Centro de Biotecnologia, Universidade Federal do Rio Grande do Sul (UFRGS), Porto Alegre, Rio Grande do Sul, Brazil; 2 Laboratório de Biologia Molecular de Cestódeos, and Laboratório de Genômica Estrutural e Funcional, Centro de Biotecnologia, Universidade Federal do Rio Grande do Sul (UFRGS), Porto Alegre, Rio Grande do Sul, Brazil; 3 Departamento de Biologia Molecular e Biotecnologia, Instituto de Biociências, Universidade Federal do Rio Grande do Sul, Porto Alegre, Brazil; 4 Biological Mass Spectrometry Laboratory, Clinical Chemistry Branch, Division of Laboratory Sciences, National Center for Environmental Health, Centers for Disease Control and Prevention, Atlanta, Georgia, United States of America; The First Affiliated Hospital of Xinjiang Medical University, CHINA

## Abstract

**Background:**

The proteins responsible for the key molecular events leading to the structural changes between the developmental stages of *Echinococcus granulosus* remain unknown. In this work, azidohomoalanine (AHA)-specific labeling was used to identify proteins expressed by *E*. *granulosus* protoscoleces (PSCs) upon the induction of strobilar development.

**Methodology/Principal Findings:**

The *in vitro* incorporation of AHA with different tags into newly synthesized proteins (NSPs) by PSCs was analyzed using SDS-PAGE and confocal microscopy. The LC-MS/MS analysis of AHA-labeled NSPs by PSCs undergoing strobilation allowed for the identification of 365 proteins, of which 75 were differentially expressed in comparison between the presence or absence of strobilation stimuli and 51 were expressed exclusively in either condition. These proteins were mainly involved in metabolic, regulatory and signaling processes.

**Conclusions/Significance:**

After the controlled-labeling of proteins during the induction of strobilar development, we identified modifications in protein expression. The changes in the metabolism and the activation of control and signaling pathways may be important for the correct parasite development and be target for further studies.

## Author Summary

In the life cycle of the parasite *Echinococcus granulosus*, hydatid cysts produce the pre-adult form, which has the ability to either differentiate into an adult worm (strobilation) or dedifferentiate into a secondary hydatid cyst. We used different protein tags that allowed for the visualization and purification of proteins produced specifically after the induction of strobilar development to identify proteins that might be involved in this process (temporally controlled and context-dependent). As a result, we found proteins that are involved in important processes during development, such as energy metabolism, control pathways and cellular communication. We believe that these results will be useful for the development of scientific approaches to controlling and preventing cystic hydatid disease.

## Introduction

The parasite *Echinococcus granulosus* is a cestode tapeworm that acts as the causative agent of cystic echinococcosis (cystic hydatid disease), one of the 17 neglected tropical diseases to be recently prioritized by the World Health Organization [1]. During its life cycle, the *E*. *granulosus* adult worm resides in the intestine of the definitive host (e.g., dogs), releasing their eggs with the host feces. Following ingestion by the intermediate host (e.g., domestic ungulates), the eggs release oncospheres that penetrate the intestinal wall and then migrate to various organs of the host. At the organ site, the oncosphere develops in the larval stage of the parasite, the hydatid cyst (metacestode). The pre-adult forms (protoscolex, PSC) are asexually formed in the cyst germinal cellular layer and liberated into the lumen of hydatid cysts [2–7]. In the cyst cavity, PSCs may remain in an inactive state for years until the structural integrity of the cyst is lost and they exhibit a dual developmental capacity. When ingested by a definitive host, PSCs sexually differentiate into fully developed, segmented adult worms in a process called strobilation. Alternately, upon hydatid cyst rupture and the release of its contents into the peritoneal cavity of an intermediate host, PSCs can dedifferentiate into secondary hydatid cysts [8].

This dual developmental capacity of the parasite and its requirement for more than one host to complete its life cycle are associated with its ability to readily respond to host environmental changes and regulate its gene expression and protein synthesis [9–11]. Transcriptional and proteomic studies have identified differentially expressed genes and proteins between the different life stages and cyst components of *E*. *granulosus* [6, 12–14]. However, the identities of the proteins responsible for key molecular events that lead to structural changes of the parasite and its transition between different developmental stages remain essentially unknown. One possible reason for this is the difficulty of indirectly associating changes in gene expression to the response from a particular stimulus. Consequently, the direct visualization and identification of newly synthesized proteins (NSPs) is useful for revealing the spatiotemporal characteristics of proteomes during development [15].

Recently, the application of bioorthogonal non-canonical amino acid tagging (BONCAT) and fluorescent non-canonical amino acid tagging (FUNCAT) have been described for the non-radioactive labeling, visualization, purification and identification of NSPs [11, 16, 17]. In BONCAT, newly synthesized proteins containing non-canonical amino acids containing either azide or alkyne moieties, such as the methionine (Met) analogue azidohomoalanine (AHA), are chemically combined with affinity tags. The alkyne or azide functional groups used in BONCAT require further purification steps, whereas FUNCAT uses fluorescent tags for *in situ* visualization. BONCAT has been used for labeling NSPs in response to different stimulus in mammalian [16, 17] and bacterial [18] cells. Moreover, BONCAT has been used in combination with FUNCAT to show NSPs in zebrafish [19]. Further adaptations have allowed the application of these methods to identifying NSPs in model organisms such as *Caenorhabditis elegans*, fruit fly and mouse [20].

Here, we report the application of FUNCAT and BONCAT followed by confocal, SDS-PAGE and MS analyses to study *E*. *granulosus* NSPs and to identify 365 AHA-labeled NSPs during the *E*. *granulosus* strobilar development *in vitro*. Some of the identified proteins have important functions in key processes for the survival and development of the parasite, such as metabolic reactions and processes involved in host/parasite relationships. The further applicability of these methods for developmental studies in parasitic flatworms is also discussed.

## Methods

### PSC collection

Hydatid cysts from *E*. *granulosus* (G1 genotype) were obtained from the naturally infected livers and lungs of cattle routinely slaughtered in a local abattoir (São Leopoldo, RS, Brazil). Finding hydatid cysts during mandatory animal inspection renders contaminated viscera unfit for human consumption, and *E*. *granulosus* contaminated livers and lungs were donated by the abattoir for use in this work. PSCs were collected by aspiration, decanted by gravity and washed several times with PBS, pH 7.4 [13]. The viability of PSCs was determined using the trypan blue exclusion test [21] and the motility was evaluated visually using an inverted light microscope. Only PSCs with viability greater than 90% were used for further analysis. The PSCs were genotyped by one-step PCR [22].

### Cultivation of PSCs and the metabolic incorporation of AHA

PSCs were cultured *in vitro* as previously described [23], with minor modifications. Briefly, PSCs were incubated at 37°C and 5% CO_2_ for 15 min with pepsin (2 mg/mL, Sigma, St. Louis, MO, USA) in Hanks’ Balanced Salt Solution (HBSS), pH 2.0. The PSCs were then washed three times with PBS containing antibiotics (100 IU/mL penicillin and 100 mg/mL streptomycin, Sigma, St. Louis, MO, USA) before being incubated with the appropriate medium. To stimulate the PSCs to undergo strobilar development (SSD), the PSC suspension was transferred to a biphasic medium. Approximately 500 PSCs were used per mL of liquid medium over the solid base. The biphasic medium contained coagulated newborn calf serum (Gibco, Auckland, NZ) as the solid phase, which was obtained by heating the serum at 76°C in a water bath for 10 to 30 min. Each 100 mL of the liquid phase consisted of 83.5 mL RPMI without Met (Gibco, Grand Island, NY, USA), 15 mL fetal bovine serum (Vitrocell, Campinas, SP, BR), 1.15 mL glucose (Merck, Loughborough, UK) 30% in 18 megaOhm water, 0.35 mL taurocholate 0.2% (Sigma, St. Louis, MO, USA) in HBSS, 100 IU/mL penicillin/streptomycin and AHA (Invitrogen, Eugene, OR, USA) in a final concentration of 50 μM. The control without stimuli for strobilar development (NSD) consisted of PSCs maintained in a monophasic medium containing RPMI without Met, 15% of fetal bovine serum and AHA (50 μM). The negative control (NC) for AHA incorporation consisted of the NSD condition without AHA. The cultures were maintained at 37°C and 5% CO2 for 24 h for the proteome analysis or 72 h for the other experiments.

### Detection of newly synthesized proteins

#### SDS-PAGE

Cell lysis and protein extraction were performed using the Click-It Metabolic Labeling Reagents for Proteins kit (Invitrogen, Eugene, OR, USA) according to manufacturer’s instructions, with modifications. Briefly, the PSCs were transferred from the culture medium to a 1.5 mL tube and washed three times with PBS. Then, 200 μL of lysis buffer (1% SDS in 50 mM Tris-HCl, pH 8.0) were added per 1500 PSCs. After 15 min of incubation on ice, the PSCs were gently homogenized using a needle and a syringe. The lysate was sonicated for 3 x 30 sec with a 60 sec interval between pulses, vortexed for 5 min and centrifuged at 13,000×*g* at 4°C for 5 min. The protein concentration was determined by fluorometry using the Qubit Protein Assay Kit (Invitrogen, Carlsbad, CA, USA), followed by precipitation with methanol/chloroform.

The proteins were solubilized in lysis buffer and 200 μg were labeled using the Click-iT Tetramethylrhodamine (TAMRA) alkyne Protein Analysis Detection Kit (Invitrogen, Eugene, OR, USA) according to the manufacturer's instructions. After labeling, the proteins were precipitated with methanol/chloroform, resuspended in Thiourea/Urea buffer (7 M urea, 2 M thiourea, 4% CHAPS) and separated in 12% SDS-PAGE. The TAMRA-labeled proteins were visualized under 300 nm ultraviolet (UV) light.

#### Confocal microscopy

To visualize the spatial distribution of AHA-labeled NSPs, whole mount PSCs were fixed at room temperature with 4% paraformaldehyde (in PBS) for 30 min. The PSCs were permeabilized with proteinase K (20 μg/mL) for 20 min, followed by two 5-min washes in PBS/0.1 M glycine, two 15-min washes in PBS/0.1% Triton X-100 and a final 10-min wash in PBS/1% BSA before conjugation with Alexa Fluor 488 Alkyne using the Click-iT Cell Reaction Buffer Kit (Invitrogen, Eugene, OR, USA). Subsequently, the DNA was stained with 50 mM 4',6-diamidino-2-phenylindole (DAPI) in PBS-Tween 0.05% containing 1% BSA for 20 min. Fluoromount (Sigma, St. Louis, MO, USA) and coverslips were used to preserve the PSCs. Images were obtained using an Olympus FluoView 1000 confocal microscope and the post-acquisition processing and analysis were performed using ImageJ (NCBI, NIH). One-Way Anova statistical analysis was performed, assuming *p*<0.05 as significant.

### Identification of newly synthesized proteins

#### Sample preparation and mass spectrometry

The PSC protein extraction was performed according to the Click-iT Protein Enrichment Kit (Invitrogen, Eugene, OR, USA) manufacturer’s instructions. Briefly, the PSCs were incubated in urea lysis buffer (8 M urea, 200 mM Tris pH 8.0, 4% CHAPS, 1 M NaCl) supplemented with protease inhibitors (Sigma, St. Louis, MO, USA) on ice for 10 min. Then, the lysate was sonicated for 3 x 30 sec with a 60 sec interval between pulses, vortexed for 5 min and centrifuged at 10,000×*g* at 4°C for 5 min. An 800 μL aliquot of supernatant was used for click chemistry reactions with alkyne agarose resin. Protein reduction with DTT at 70°C and alkylation with iodoacetamide at room temperature were performed before the resin was washed with 5 x 2 mL SDS Wash Buffer, 10 x 2 mL 8 M urea and 10 x 2 mL 20% acetonitrile for the stringent removal of non-specifically bound proteins. The resin-bound proteins were transferred to a clean tube and digested with trypsin (10 μL of 0.1 μg/μL, Promega, Madison, WI, USA) in approximately 200 μL of digestion buffer (100 mM Tris, 2 mM CaCl_2_, 10% acetonitrile). After a 5-min centrifugation at 1,000×*g*, the supernatant was treated with 2% acetonitrile, acidified with trifluoroacetic acid and desalted in HLB cartridges (Waters, Milford, MA, USA). The peptides were then eluted with 50% acetonitrile/0.1% TFA, quantified using a NanoDrop (Thermo Fisher Scientific, Waltham, MA, USA) at 205 nm and lyophilized in a SpeedVac concentrator.

The peptides were reconstituted using 0.1% formic acid (Thermo Scientific, Rockford, IL) in water (Burdick and Jackson, Muskegon, MI), loaded onto a nanoAcquity UPLC system (Waters Corporation, Milford, MA) and separated using a gradient elution. The mobile phase solvents consisted of (solvent A) 0.1% formic acid in water and (solvent B) 0.1% formic acid in acetonitrile (Burdick and Jackson). The gradient flow was set at 300 nl/min. The profile consisted of a hold at 5% B for 5 min, followed by a ramp up to 35% B over 25 min, then a ramp up to 95% B in 5 min, a hold at 95% for 5 min before returning to 5% B in 5 min and re-equilibration at 5% B for 20 min. After chromatography, the peptides were introduced into an Orbitrap Elite tandem mass spectrometer (Thermo Scientific, San Jose, CA). A 2.0 kV voltage was applied to the nano-LC column. The mass spectrometer was programmed to perform data-dependent acquisition by scanning the mass range from mass-to-charge (m/z) 400 to 1600 at a nominal resolution setting of 60,000 for parent ion acquisition in the Orbitrap. For the MS/MS analysis, the mass spectrometer was programmed to select the top 15 most intense ions with two or more charges. The experiment was performed in two biological and two technical replicates. Each biological sample was composed of PSCs collected from one hydatid cyst.

#### Data analysis

The MS/MS raw data were processed using msConvert (ProteoWizard, version 3) [24], and the peak lists were exported in the Mascot Generic Format (.mgf). The MS/MS data were analyzed using Mascot Search Engine (Matrix Science, version 2.3.02) against a local *E*. *granulosus* database (21764 sequences) containing the deduced amino acid sequences from the genome annotation available on GeneDB [25] and the Chinese National Human Genome Center at Shanghai (CHGCS) [6]. The search parameters included a fragment ion mass tolerance of 1 Da and a peptide ion tolerance of 10 ppm. Carbamidomethylation was specified as a fixed modification, whereas the oxidation of methionine was specified as a variable modification.

Scaffold (Proteome Software Inc., version 4.4.1) was used to validate the peptide and protein identifications. The peptide identifications were accepted if they could be established at greater than 95.0% probability as assigned by the Peptide Prophet algorithm [26]. The protein identifications were accepted if they could be established at greater than 99% probability as assigned by the Protein Prophet algorithm [27] and contained at least 2 identified peptides. The false discovery rate, FDR (Decoy), was 0.0% for proteins and peptides. The normalized spectral abundance factor (NSAF) [28] was calculated for each protein, and the quantitative differences were statistically analyzed using Student’s t-test in Scaffold. The differences with p-values lower than 0.05 were considered statistically significant. Proteins with p-value < 0.05 were selected for the hierarchical clustering analysis. A heat map graphical representation was performed in Perseus software (MaxQuant, version 1.5.1.6.) [29]. Protein NSAFs were normalized by their Z-scores and clustered using the Euclidean distance method with average linkages.

The eggNOG database (version 4.1, http://eggnogdb.embl.de/#/app/home) [30] was used to acquire the functional annotation for the identified proteins.

## Results

We have established an *in vitro* model of *E*. *granulosus* PSC strobilar development under low potential sources of Met and conditions that promote the efficient incorporation of AHA. This treatment has kept the characteristic morphological changes of strobilar development without affecting viability (S1 and S2 Figs; S1 Text). In addition, we analyzed the genomic data for *E*. *granulosus* to confirm the feasibility of AHA labeling because proteins with non-terminal Met residues are required for this purpose (S1 Text).

### Detection of newly synthesized proteins detection

**Fig 1:**
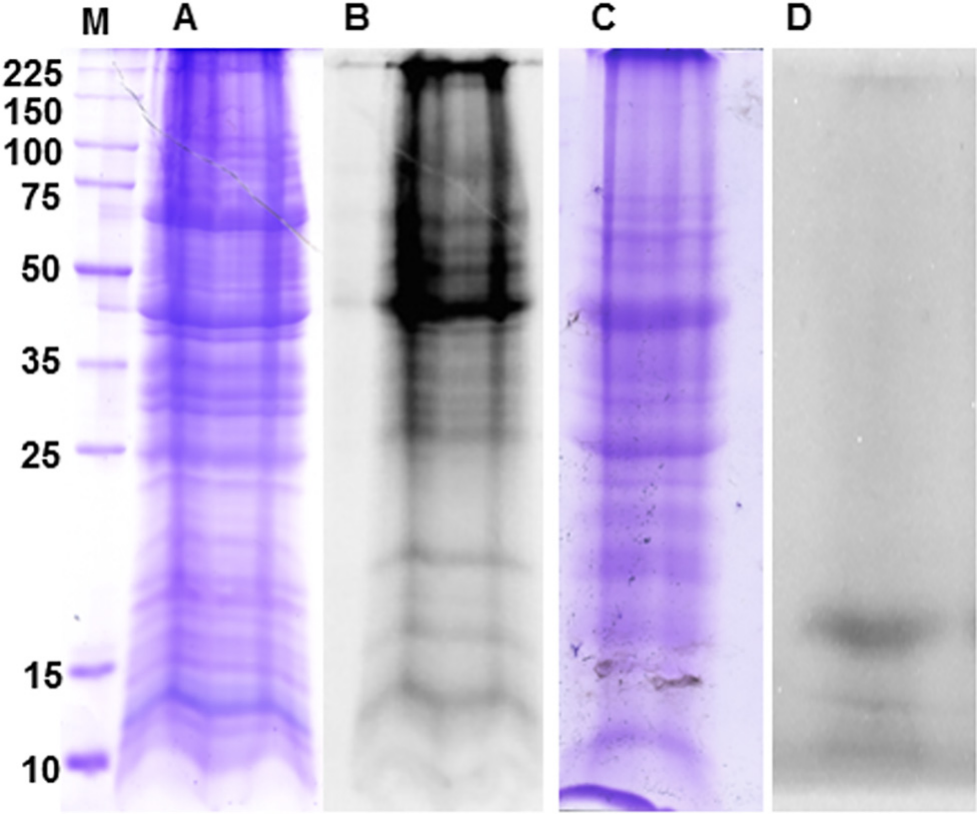
Detection of NSPs from *in vitro* cultured *E*. *granulosus* PSCs. The coomassie-stained proteins and UV detected TAMRA-labeled NSPs from the PSCs incubated for 72 h in the presence (A and B) or absence (C and D) of AHA, respectively.

The proteins synthesized by *E*. *granulosus* PSCs after 72 h of cultivation in the presence of AHA (SSD) were labeled with TAMRA and visualized under UV to verify their electrophoretic profiles. The pattern of bands in the total extract of PSCs was assessed by inspection of the gels stained with coomassie blue (Fig 1A). The NSPs of the PSCs could be visualized by UV in the same gel. The analysis of the NSPs revealed a complex but lower number of distinct banding patterns that ranged from 10 to 225 kDa (Fig 1B). In the negative control condition without AHA, the total proteins were visualized with coomassie blue (Fig 1C), but no band corresponding to NSPs was visualized under UV (Fig 1D) except for a non-incorporated TAMRA residual band, indicating the high specificity of the reaction.

**Fig 2:**
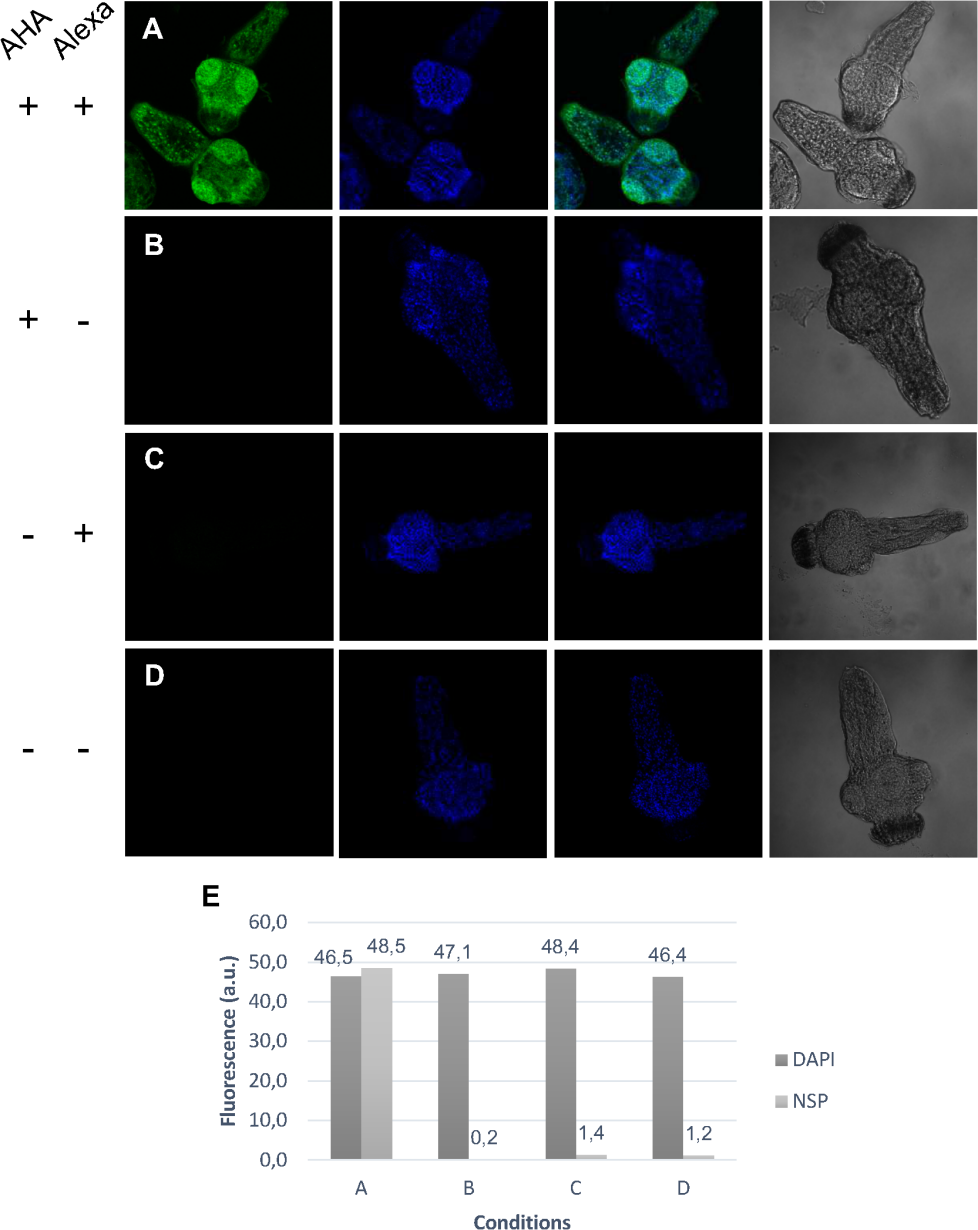
*In toto* visualization of proteins synthesized by *E*. *granulosus* PSCs induced to strobilar development. (A) NSPs were specifically visualized in the presence of AHA and Alexa Fluor 488 Alkyne. The other images represent the DAPI nuclei staining, the bright field merges of DAPI and Alexa Fluor 488 antibody staining and the bright field images. (B) AHA+/Alexa-, (C) AHA-/Alexa+ and (D) AHA-/Alexa- did not show significant fluorescence or autofluorescence (400x). (E) The quantification of NSP (Alexa Fluor 488) and nucleic acid regions (DAPI) fluorescence. a.u., arbitrary units.

To evaluate whether the AHA-labeling methodology could be applied to identifying NSPs from *in toto* PSCs and to verify whether the labeled proteins were associated with a particular site or structure in PSCs undergoing strobilation (SSD), Alexa Fluor 488-labeled AHA-containing NSPs were analyzed in whole mount PSCs by confocal microscopy after 72 h of *in vitro* culture (Fig 2). The fluorescence signal corresponding to the labeled NSPs was widely distributed and could be detected all over the PSCs (Fig 2A), though there was a possible correlation between the NSPs and the suckers. In contrast, no NSP-associated fluorescence could be detected in the control experiments. The levels of DAPI fluorescence remained stable under the different conditions (Fig 2B–2D). The estimated fluorescence for the NSP-Alexa Fluor and DAPI are shown in Fig 1E.

### Identification of newly synthesized proteins

**Fig 3:**
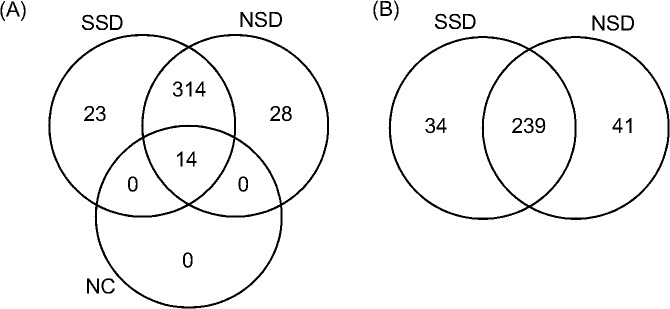
Proteins identified in *E*. *granulosus* PSCs after the induction of strobilar development. Venn diagram showing the identified proteins in the AHA and control samples. (A) Exclusive proteins identified in SSD and CSD. (B) Differentially expressed proteins.

We next explored the identities of the NSPs expressed in PSCs undergoing strobilation over a 24-h time window. As we searched for proteins involved in the strobilation process, we chose a shorter culture time because longer times may dilute the importance of these molecules. Scaffold was used to validate 365 non-redundant proteins in the AHA samples (Fig 3). In the non-AHA samples, we found 14 proteins that were excluded from subsequent analyzes. The purified NSPs from PSCs either with strobilation stimuli (SSD) or without strobilation stimuli (NSD) were identified by LC-MS/MS, which allowed for the identification of 248 and 275 proteins, respectively. A list of the newly synthesized proteins identified in both samples is provided in S1 Table.

We found 51 proteins (23 SSD and 28 NSD) in only one of the conditions studied in at least two replicates. We normalized the other 233 identified proteins by NSAF and applied Student’s t-test to find the differentially expressed proteins. 75 proteins were considered to be differentially expressed, with 34 considered to be more highly expressed in SSD and 41 more highly expressed in NSD (Table 1).

**Table 1:** Differentially expressed proteins.

Functional annotation^a^	Identified proteins^b^ ^,^ ^c^	Accession Number	NSAF SSD^d^	NSAF NSD^e^	P-value	Fold change
SSD proteins						
Amino acid transport and metabolism	Glutamate dehydrogenase mitochondrial	EgrG_000604400.1	0,00218163	0	-	-
	2 amino 3 ketobutyrate coenzyme A ligase	EgrG_000107200.1	0,00500288	0	-	-
Cytoskeleton	Myosin regulatory light chain A, smooth adductor muscle	gi|576695936	0,14420750	0,11952750	0,0027	1,2
	Actin cytoplasmic type 5	EgrG_000190400.1	0,01251020	0,00709483	0,034	1,8
	Myosin heavy chain, striated muscle	gi|576698220	0,02525800	0,01340450	0,0017	1,9
	Dynein light chain 1, cytoplasmic	gi|576696895	0,13540500	0,06898675	0,0043	2,0
	Myosin regulatory light chain 2 smooth muscle	EgrG_000041600.1	0,02763825	0,01373125	0,049	2,0
	Actin cytoplasmic A3	EgrG_000406900.1	0,01701225	0,00795388	0,0055	2,1
Energy production and conversion	ATP synthase subunit beta mitochondrial	EgrG_000752000.1	0,00210028	0	-	-
	NADP dependent malic enzyme	EgrG_001145700.1	0,00237255	0	-	-
	Pyruvate dehydrogenase E1 component subunit	EgrG_000590700.1	0,00304668	0	-	-
	NADH cytochrome b5 reductase 3	EgrG_000865900.1	0,00339048	0	-	-
	Aconitate hydratase mitochondrial	EgrG_000158240.1	0,00365168	0	-	-
	Succinate dehydrogenase ubiquinone	EgrG_000422600.1	0,00774378	0	-	-
	Pyruvate dehydrogenase	EgrG_000956200.1	0,00862753	0	-	-
	Cytosolic malate dehydrogenase	EgrG_000417100.1	0,05691600	0,03523775	0,036	1,6
	Succinate dehydrogenase (ubiquinone) iron sulfur	EgrG_000416100.1	0,04523500	0,02794375	0,00045	1,6
	Phosphoenolpyruvate carboxykinase [GTP]	gi|576694081	0,07943825	0,04717400	< 0,00010	1,7
	Citrate synthase	EgrG_001028500.1	0,02500050	0,01324200	0,00059	1,9
Function unknown	Hypothetical protein	gi|576695609	0,00378138	0	-	-
	RNA binding protein EWS	EgrG_001020700.1	0,01534125	0,00991710	0,011	1,5
	Expressed conserved protein	EgrG_000696700.1	0,00851018	0,00211125	0,015	4,0
	Hypothetical protein	gi|576692667	0,01587075	0,00369325	0,023	4,3
Intracellular trafficking, secretion, and vesicular transport	Charged multivesicular body protein	gi|576693582	0,01026600	0	-	-
	Charged multivesicular body protein 4B	EgrG_001133200.1	0,02666625	0,01683500	0,041	1,6
	Clathrin light chain	EgrG_000925600.1	0,01228048	0,00764348	0,025	1,6
	Charged multivesicular body protein 5	EgrG_001063800.1	0,01977925	0,00958983	0,011	2,1
	Charged multivesicular body protein 3	EgrG_000238400.1	0,02452800	0,00300075	0,0042	8,2
Nucleotide transport and metabolism	Nucleoside diphosphate kinase A 2	gi|576695995	0,00894200	0	-	-
	Dihydropyrimidinase	EgrG_000953400.1	0,01286375	0,00754355	0,005	1,7
Post-translational modification, protein turnover, and chaperones	Heat shock 70 kDa protein 4	EgrG_000938600.1	0,00130175	0	-	-
	26S proteasome regulatory subunit T3	EgrG_000223000.1	0,00212315	0	-	-
	T-complex protein 1 subunit epsilon	gi|576697091	0,00235945	0	-	-
	Calreticulin	gi|576693417	0,00287313	0	-	-
	Activator of 90 kDa heat shock protein ATPase	EgrG_000241900.1	0,00451208	0	-	-
	T-complex protein 1 subunit zeta	gi|576693013	0,00625755	0	-	-
	26S proteasome regulatory subunit N11	EgrG_001166800.1	0,00680358	0	-	-
	Heat shock protein	gi|576694604	0,00444963	0,00395170	0,0058	1,1
	Transitional endoplasmic reticulum ATPase	EgrG_000471600.1	0,03403750	0,02595500	0,024	1,3
	Ubiquitin conjugating enzyme	EgrG_000616800.1	0,04567950	0,03198050	0,0043	1,4
	Calnexin	EgrG_000875100.1	0,00900948	0,00611455	0,027	1,5
	WAP, Kazal, immunoglobulin, Kunitz and NTR domain-containing protein 2	gi|576694291	0,03321825	0,02210675	0,042	1,5
	Major egg antigen p40	EgrG_000212700.1	0,06261775	0,03261750	0,00025	1,9
	Ubiquilin-1	gi|576694418	0,01723425	0,00737003	0,00038	2,3
	Heat shock protein 60	EgrG_001190900.1	0,11921750	0,04692450	< 0,00010	2,5
	Protein AHNAK2	EgrG_000760400.1	0,01505950	0,00299023	< 0,00010	5,0
	T complex protein 1 subunit gamma	EgrG_000872100.1	0,00943840	0,00080528	0,0044	11,7
RNA processing and modification	RNA recognition motif RRM domain containing protein	EgrG_000264300.1	0,00222925	0	-	-
	Spliceosome RNA helicase BAT1	EgrG_000546900.1	0,00705580	0,00101435	0,0082	7,0
Signal transduction mechanisms	Calcium binding protein P22	EgrG_000447500.1	0,04368925	0,02742450	0,038	1,6
Transcription	Serrate RNA effector molecule	gi|576694739	0,00042201	0	-	-
	SNW domain-containing protein	gi|576692125	0,00238970	0	-	-
	Transcription elongation regulator 1	EgrG_000862700.1	0,00334500	0,00144693	0,0082	2,3
	Elongation factor 1 delta	EgrG_000517100.1	0,01624900	0,00681798	< 0,00010	2,4
Translation, ribosomal structure and biogenesis	Aminoacyl tRNA synthase complex interacting	EgrG_001100200.1	0,00423773	0	-	-
	40S ribosomal protein AS	EgrG_000720100.1	0,02209325	0,00692395	0,00023	3,2
	Eukaryotic translation initiation factor 3	EgrG_000068000.1	0,00730010	0,00151090	0,018	4,8
**NSD proteins**						
Carbohydrate transport and metabolism	Triosephosphate isomerase	EgrG_000416400.1	0,01662075	0,02560950	0,044	1,5
	Fructose 16 bisphosphate aldolase	EgrG_000905600.1	0,03377200	0,05451625	0,00016	1,6
Cell cycle control, cell division, chromosome partitioning	Rab11 family-interacting protein	gi|576698836	0	0,0016588	-	-
	Structural maintenance of chromosomes protein 2	EgrG_000602100.1	0,0073003	0,0101055	0,0069	1,4
	Translationally controlled tumor protein	EgrG_000058000.1	0,0060565	0,0223265	0,012	3,7
Cell wall/membrane/envelope biogenesis	Ankyrin-3	gi|576691831	0	0,00062894	-	-
Cytoskeleton	Protein kinase C and casein kinase substrate in	EgrG_001090800.1	0	0,00113730	-	-
	Ezrin	EgrG_000517000.1	0	0,00118645	-	-
	Troponin i	EgrG_000734700.1	0	0,00336850	-	-
	Microtubule associated protein RP:EB family	EgrG_000637300.1	0	0,00603648	-	-
	Dynein light chain	EgrG_000071300.1	0	0,02840825	-	-
	Myosin essential light chain, striated adductor muscle	gi|576700259	0,11973250	0,15936500	0,00025	1,3
	Spectrin alpha chain	gi|576696380	0,00739963	0,00999265	0,0081	1,4
	Myophilin	gi|576693412	0,01986200	0,02702125	0,03	1,4
	Tubulin alpha-1C chain	gi|576701165	0,01033978	0,01517000	0,028	1,5
	Tropomyosin-2	gi|576693326	0,00569973	0,00891180	0,035	1,6
	Actin modulator protein	EgrG_000882500.1	0,01606350	0,02763525	0,0032	1,7
	Gamma aminobutyric acid receptor associated	EgrG_001158000.1	0,01022620	0,02080425	0,0037	2,0
	Hematopoietic lineage cell specific protein	EgrG_000936900.1	0,00072758	0,00487913	0,0095	6,7
	Cofilin/actin-depolymerizing factor	gi|576697710	0,00215878	0,02190575	0,0012	10,1
Function unknown	Expressed conserved protein	EgrG_000203800.1	0	0,00223970	-	-
	Hypothetical protein	gi|576699770	0	0,00279715	-	-
	Guanine nucleotide binding protein subunit	EgrG_000200300.1	0	0,00626868	-	-
	Cystatin B stefin B	EgrG_000159200.1	0	0,01504050	-	-
	Expressed conserved protein	EgrG_001061900.1	0,00958830	0,01356800	0,03	1,4
	Hypothetical protein	gi|576700828	0,00681093	0,01128425	0,0049	1,7
	Sj Ts4 protein	EgrG_000393000.1	0,01064943	0,02080450	0,0028	2,0
	Hypothetical protein	gi|576692345	0,00112804	0,00235835	0,0058	2,1
	Programmed cell death 6 interacting protein	EgrG_000997550.1	0,00064300	0,00363378	0,04	5,7
	Hypothetical protein	gi|576690197	0,00036507	0,00254528	0,0052	7,0
Inorganic ion transport and metabolism	Na:K ATPase alpha subunit	EgrG_000342600.1	0,00232473	0,00436733	0,018	1,9
Intracellular trafficking, secretion, and vesicular transport	Sorting nexin	EgrG_000922200.1	0	0,01089525	-	-
	Annexin A6	gi|576697441	0,00147718	0,00481598	0,017	3,3
Post-translational modification, protein turnover, and chaperones	Ubiquitin supergroup	EgrG_001180300.1	0	0,01742975	-	-
	Heat shock protein 71 kDa protein	EgrG_001085100.1	0,02868700	0,03494425	0,048	1,2
	Stress induced phosphoprotein 1	EgrG_000264900.1	0,03160575	0,03906600	0,032	1,2
	Thioredoxin peroxidase	EgrG_000791700.1	0,05674150	0,07183575	0,0053	1,3
	Dnaj subfamily A	EgrG_000101800.1	0,03477350	0,04575175	0,0057	1,3
	Dnaj subfamily B	EgrG_000614200.1	0,03305725	0,04662200	0,0029	1,4
	Calreticulin	gi|576693418	0,00577333	0,00862368	0,033	1,5
	Calpain	EgrG_000719700.1	0,00653558	0,01014048	0,017	1,6
	Ubiquitin-conjugating enzyme E2 L3	gi|576700841	0,00335100	0,01493200	0,031	4,5
	Tumor protein D52	EgrG_000949820.1	0,00238700	0,01167500	0,0081	4,9
Replication, recombination and repair	Phosphatase 2A inhibitor I2PP2A	EgrG_000465500.1	0,02583550	0,03972525	0,0014	1,5
RNA processing and modification	RNA binding protein fox 1 3	EgrG_000063400.1	0	0,00132858	-	-
	ATP dependent RNA helicase DDXx	EgrG_000098400.1	0	0,00231058	-	-
	U2 small nuclear RNA auxiliary factor 2	EgrG_000625400.1	0	0,00292313	-	-
	Polyadenylate binding protein	EgrG_000689000.1	0,00971535	0,01186025	0,036	1,2
	Heterogeneous nuclear ribonucleoprotein A1	EgrG_000807100.1	0,03108225	0,04304250	0,00081	1,4
	Fragile X mental retardation syndrome-related protein	gi|576697665	0,00311460	0,00595435	0,026	1,9
	Heterogeneous nuclear ribonucleoprotein K	gi|576694356	0,00188858	0,00516005	0,0063	2,7
Secondary metabolites biosynthesis, transport, and catabolism	ES1 protein mitochondrial	EgrG_000999800.1	0	0,00349188	-	-
Signal transduction mechanisms	SH2 motif	EgrG_000343500.1	0	0,00097938	-	-
	Tyrosine protein kinase otk	EgrG_000212300.1	0	0,00210218	-	-
	Nuclear migration protein nudc	EgrG_000463400.1	0	0,00241293	-	-
	PDZ domain containing protein GIPC3	EgrG_000190700.1	0	0,00396823	-	-
	Na:H exchange regulatory cofactor NHE RF2	EgrG_000743800.1	0	0,00443958	-	-
	Serine:threonine protein phosphatase PP1 gamma	EgrG_000779500.1	0	0,00607725	-	-
	Transforming protein RhoA	EgrG_000246600.1	0	0,00812860	-	-
	Neuronal calcium sensor	EgrG_000186300.1	0	0,01437175	-	-
	Titin	EgrG_000610500.1	0,00034956	0,00063859	0,009	1,8
	Universal stress protein	EgrG_000873800.1	0,00811793	0,02731575	0,00055	3,4
	Arfaptin 2	EgrG_000192300.1	0,00156715	0,00686460	0,039	4,4
	Endophilin B2	EgrG_000060900.1	0,00288233	0,01315925	0,0056	4,6
Transcription	Transcriptional repressor P66-beta	gi|576694405	0	0,00156535	-	-
	Transcription factor AP 4	EgrG_000768400.1	0	0,00621390	-	-
	Actin-depolymerizing factor 2	gi|576697711	0,00135023	0,00318298	0,03	2,4
Translation, ribosomal structure and biogenesis	40S ribosomal protein S28	EgrG_001150800.1	0	0,03077775	-	-
	Ribosomal protein LP1	EgrG_001195100.1	0,03494200	0,04921800	0,015	1,4

The proteins that were identified as exclusive in at least two replicates or the proteins that were differentially expressed with significant T-Test values (*p*≤0.05) are presented.

^a^Functional classification determined by eggNOG.

^*b*^Protein accession numbers according to GeneDB (www.genedb.org/).

^*c*^Protein accession numbers according to NCBI (www.ncbi.nlm.nih.gov/).

^d^Average NSAF for SSD replicates.

^e^Average NSAF for NSD replicates.

**Fig 4:**
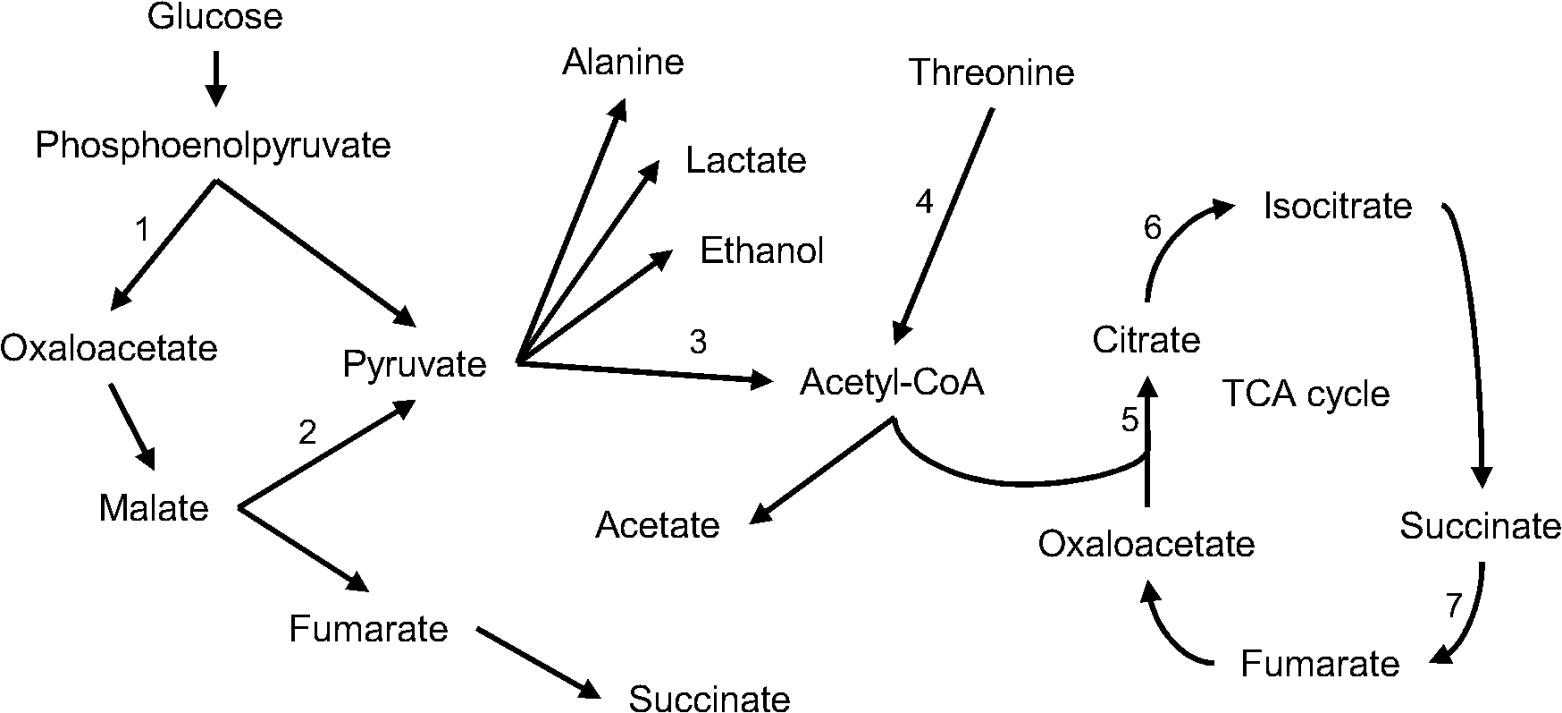
Metabolism in *E*. *granulosus* PSCs after induction of the strobilar stage. A representative schematic of the metabolic pathways that may be active during the strobilar development of PSCs. These reactions comprise the degradation of carbohydrates to phosphoenolpyruvate and the production of pyruvate and succinate.1) Phosphoenolpyruvate carboxykinase; 2) NADP-dependent malic enzyme; 3) Pyruvate dehydrogenase; 4) 2 amino 3 ketobutyrate coenzyme A ligase; 5) Citrate synthase; 6) Aconitate hydratase mitochondrial; and 7) Succinate dehydrogenase ubiquinone.

In SSD, several proteins are involved in metabolic reactions (Fig 4) and may indicate how the parasite obtains energy during its development into the adult worm. A feasible metabolic pathway for *E*. *granulosus* involves phosphoenolpyruvate, which is carboxylated to give oxaloacetate. Oxaloacetate is reduced to malate, which is then either oxidatively decarboxylated to pyruvate or reduced to succinate. Moreover, pyruvate can be converted into acetyl-CoA, which can participate in the tricarboxylic acid cycle or be converted into acetate.

**Fig 5:**
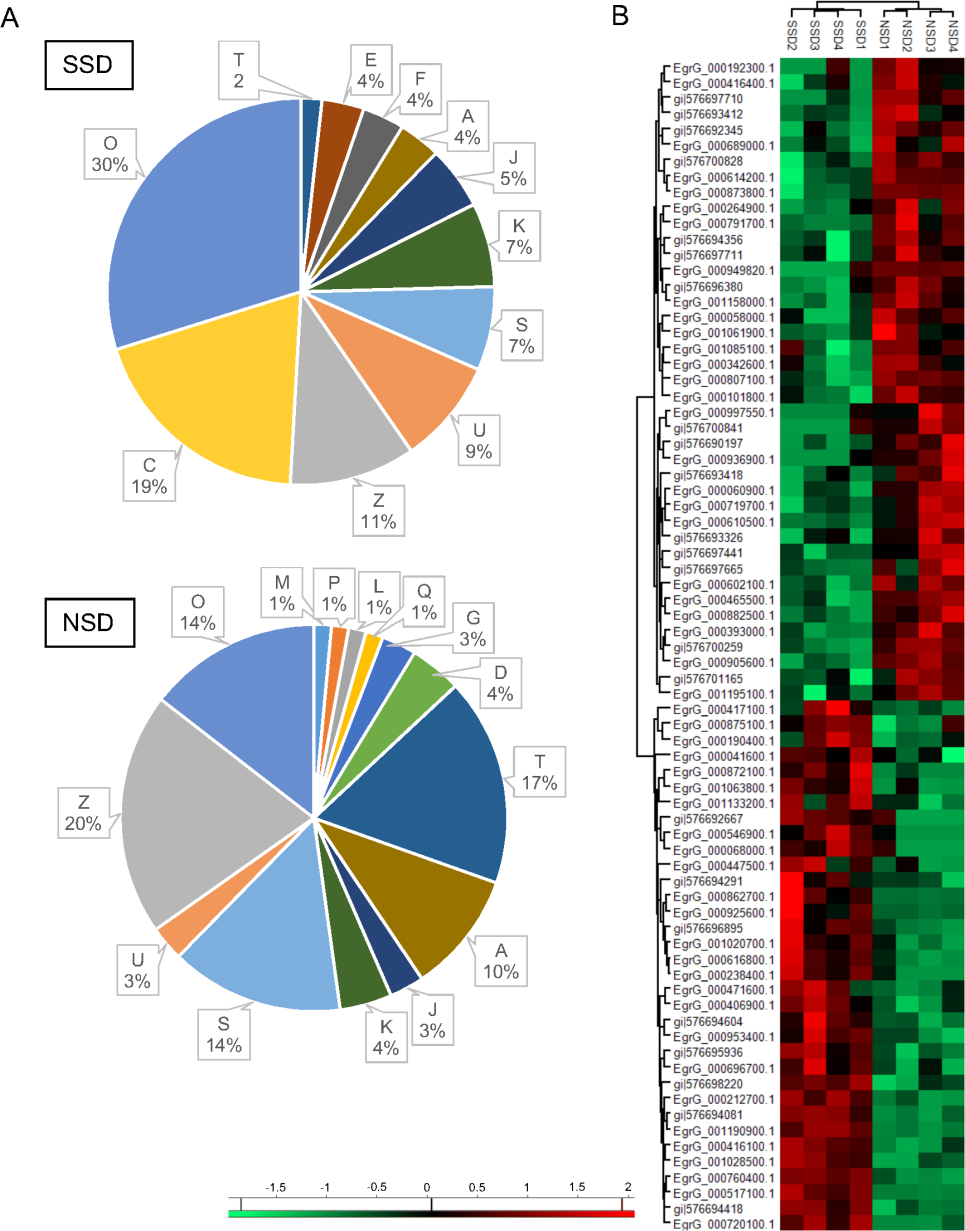
Comparative analysis of NSPs from *E*. *granulosus* PSCs after the induction of strobilar development. (A) Functional categories of total identified NSPs. Percentages of identified proteins in each functional category are indicated. (O) Post-translational modification, protein turnover, and chaperones; (C) Energy production and conversion; (Z) Cytoskeleton; (U) Intracellular trafficking, secretion, and vesicular transport; (S) Function unknown; (K) Transcription; (J) Translation, ribosomal structure and biogenesis; (A) RNA processing and modification; (F) Nucleotide transport and metabolism; (E) Amino acid transport and metabolism; (T) Signal transduction mechanisms; (D) Cell cycle control, cell division, chromosome partitioning; (G) Carbohydrate transport and metabolism; (Q) Secondary metabolites biosynthesis, transport, and catabolism; (L) Replication, recombination and repair; (P) Inorganic ion transport and metabolism; (M) Cell wall/membrane/envelope biogenesis. The distribution of level 3 biological processes for SSD and NSD-exclusive and up-regulated proteins. (B) A heat map from NSPs with high (red) or low (green) expression levels between the SSD and NSD groups.

A functional annotation of the NSPs is presented in Table 1 (data summarized in Fig 5A). The most representative terms in the SSD were related to post-translational modification, protein turnover, and chaperones (O, 30%), energy production and conversion (C, 19%), cytoskeleton (Z, 11%), intracellular trafficking, secretion, and vesicular transport (U, 9%) and transcription (K, 7%). In the NSD condition, the most representative functions were cytoskeleton (Z, 20%), signal transduction mechanisms (T, 17%), post-translational modification, protein turnover, and chaperones (O, 15%) and RNA processing and modification (A, 10%). A hierarchical clustering of the up-regulated proteins listed in Table 1 was performed using the Z-score calculation on NSAF, and the results were represented as a heat map (Fig 5B). Two main clusters of the NSPs during the strobilar stimuli showing high (red) or low (green) expression.

## Discussion

Techniques that allow for the selective labeling of molecular targets are powerful tools for understanding the molecular pathways involved in the strobilation process and the identification of key proteins that are activated in response to specific stimuli. The high specificity of AHA-labeling has been proven to be non-toxic as it does not alter the global protein synthesis rates or cause significant protein misfolding or degradation [16, 19, 20, 31]. Furthermore, AHA is incorporated exclusively into NSPs without interfering with preexisting proteins.

Ethical and practical difficulties in undertaking *in vivo* studies bring up the necessity to develop *in vitro* systems. Thus, we have successfully obtained an *in vitro* model of *E*. *granulosus* PSCs undergoing strobilar development under low potential sources of Met and conditions that facilitate the efficient incorporation of AHA. The genomic data for *E*. *granulosus* were also analyzed and confirm the feasibility of AHA labeling because proteins with non-terminal Met residues are required for this purpose (S1 Text). Another factor contributing to the success of AHA-labeling is that the genome of this parasite showed no machinery for the endogenous synthesis of Met [6].

SDS-PAGE and confocal microscopy were useful in detecting the incorporation of AHA. The confocal microscopy showed a possible correlation between the NSPs and PSCs suckers. In *Mesocestoides corti*, the apical massif is a polynucleated cell mass that differentiates into several cell types [32]. This structure is a part of the tegmental syncytium and is located at the top of the scolex, next to the suckers. Although studies using DNA labeling in *E*. *granulosus* have not demonstrated the existence of a proliferation site in this region [33], the possible correlation between the NSPs and the suckers may correspond to an increased protein synthesis site.

The expression pattern identified by proteomic analysis of NSPs from PSC without stimuli for strobilar development revealed proteins involved in basic cellular functions, such as metabolic processes, regulation of biological processes and cellular component organization. In this sample, we have identified proteins related to transcription, translation and cytoskeletal.

In contrast, proteomic analysis of NSPs from SSD samples indicated changes in parasite metabolism and this has been reported with development [34–36] In more advanced stages of worm development, there is a shift from cytosolic to mitochondrial metabolism, which tends to produce more acetate and succinate, two end products with a higher energy yield than lactate [36–38]. Thereby, changes in energy-producing pathways associated with maturation may be essential for both the correct progression of parasite life cycle as well as survival.

We found a SNW domain-containing protein that is a member of the SNW gene family. Human SNW encodes a transcription coactivator that can interact with vitamin D receptor (VDR) and retinoid X receptor (RXR) [39]. It is believed that VDR and RXR may play key roles in stimulating PSCs to develop into adult worms [6, 40]. The binding and activation of these receptors by bile acid salts regulates the expression of genes involved in differentiation, development, homeostasis and metabolism. Therefore, finding an SNW protein may indicate the presence of an active state of VDR/RXR in SSD, which is plausible given these findings.

We also found three charged multivesicular body proteins that are components of the endosomal sorting complex required for transport III (ESCRT-III) [41–43]. ESCRT-III participates in the degradation of the surface receptor proteins, the formation of endocytic multivesicular bodies and the down-regulation of several signaling pathways. We also identified a clathrin light chain, a subunit of clathrin that participates in several membrane traffic pathways [44–46]. Extracellular vesicles are derived from the multivesicular body and act in host/parasite relationships and cell–cell signaling [47, 48]. Although this is a preliminary result for *E*. *granulosus*, it is encouraging to find proteins related to these functions. This cell-cell communication via exosome-like vesicles has been related to sexual differentiation, survival and population density [49].

Interestingly, a comparison of the SSD up-regulated proteins showed no apparent correlation with the previously published RNAseq data [6]. However, RNAseq data includes only expression profiles from either pepsin-activated PSCs or adult worms collected from dogs, with no data available for the transition between these two stages. Therefore, we believe that this only reinforces the importance of our experimental approach in the attempt to identify early molecular events that are triggered by a developmental stimulus.

This is the first report of an efficient labeling and identification of NSPs with AHA in flatworms, which provides an interesting tool for use in the search for regulatory molecules in *E*. *granulosus* and other parasitic organisms. The temporally controlled and context-dependent labelling of synthesized proteins allow the association between molecular changes and the processes occurring during induction of strobilation. Whereas the steps of induction between different stages play a central role to the correct development of the parasite, the knowledge of such processes can have great value to the improvement of new disease control strategies. Although there is vaccine for the intermediate host [50], the WHO recommends that other stages should also be targeted for intervention [1], which would make more efficient control. Still, considering that regulatory processes may be conserved among different helminths, the results obtained here can serve as a starting point for control studies of other parasites.

## Supporting Information

S1 Fig *In vitro* induction of strobilar development in *E*. *granulosus* PSCs. PSCs after pepsin treatment (A) and after 3 days in culture without stimuli for strobilar development (B). After one week, NSD worms present a small number of calcareous corpuscles (C). PCSs after 3 days in complete biphasic medium (D). After 5 days, calcareous corpuscles are much reduced and excretory canals (e) become evident (E) and, after one week, posterior excretory bladder (bl) is also visible (F) Magnification, 100x (A, B and D) and 200x (C, E and F).
(TIF)

S2 Fig AHA is incorporated in proteins synthesized by *E*. *granulosus* PSCs. After incubation with 0–100 μM AHA for 12−72 h, the biotin-labeled AHA-containing NSPs were detected by immunoblot (A) and quantified (B) by comparing the intensities of the sample dots and the biotinylated Bovine Serum Albumin standard dots.
(TIF)

S1 Table NSPs identified in the *E*. *granulosus* PSCs. The complete list of peptides and proteins normalized by NSAF is shown. The qualitative profiles for Excl SSD or Excl NSD (protein exclusive to SSD or NSD, respectively), SSD-1R or NSD-1R (proteins identified in just one replicate of SSD or NSD, respectively), or NC-excluded (proteins identified in the negative control and excluded from analysis) or the quantitative profiles for SSD (SSD high, NSD low) or NSD (SSD low, NSD high) with their respective T-Test values are also shown.^*a*^Protein accession numbers according to GeneDB (www.genedb.org/).^*b*^Protein accession numbers according to NCBI (www.ncbi.nlm.nih.gov/).
(XLSX)

S1 Text Supplemental experiments. Additional methods and results used in genomic data analysis, PCS cultivation and dot blot assays.
(DOCX)

## References

[1] WHO. Neglected tropical diseases [cited 2015 04: 08]. Available from: **http://www.who.int/neglected_diseases/diseases/en/**.

[2] Moro P, Schantz PM. Echinococcosis: a review. Int J Infect Dis. 2009;13(2):125–33. 10.1016/j.ijid.2008.03.037 . 18938096

[3] Zhang W, McManus DP. Recent advances in the immunology and diagnosis of echinococcosis. FEMS Immunol Med Microbiol. 2006;47(1):24–41. 10.1111/j.1574-695X.2006.00060.x . 16706785

[4] Kazemi Moghadam Kakhki Z, Ghaffarifar F, Khalilpour A, Abdul Aziz F, Saadatnia G, Noordin R. IgG4 detection of Echinococcus granulosus paramyosin: a useful diagnostic test for human hydatidosis. Clin Vaccine Immunol. 2013 10.1128/CVI.00019-13 . 23365208 PMC3623400

[5] Thompson RC, Lymbery AJ. Echinococcus: biology and strain variation. Int J Parasitol. 1990;20(4):457–70. . 2210939 10.1016/0020-7519(90)90193-q

[6] Zheng H, Zhang W, Zhang L, Zhang Z, Li J, Lu G, et al The genome of the hydatid tapeworm Echinococcus granulosus. Nat Genet. 2013 10.1038/ng.2757 . 24013640

[7] Galindo M, Paredes R, Marchant C, Miño V, Galanti N. Regionalization of DNA and protein synthesis in developing stages of the parasitic platyhelminth Echinococcus granulosus. J Cell Biochem. 2003;90(2):294–303. 10.1002/jcb.10640 . 14505346

[8] Zhang WB, Jones MK, Li J, McManus DP. Echinococcus granulosus: pre-culture of protoscoleces in vitro significantly increases development and viability of secondary hydatid cysts in mice. Exp Parasitol. 2005;110(1):88–90. 10.1016/j.exppara.2005.02.003 . 15804383

[9] Huang HC, Yao LL, Song ZM, Li XP, Hua QQ, Li Q, et al Development-specific differences in the proteomics of Angiostrongylus cantonensis. PLoS One. 2013;8(10):e76982 10.1371/journal.pone.0076982 24204717 PMC3808366

[10] Somasekharan SP, Stoynov N, Rotblat B, Leprivier G, Galpin JD, Ahern CA, et al Identification and quantification of newly synthesized proteins translationally regulated by YB-1 using a novel Click-SILAC approach. J Proteomics. 2012;77:e1–10. 10.1016/j.jprot.2012.08.019 . 22967496

[11] Dieterich DC, Lee JJ, Link AJ, Graumann J, Tirrell DA, Schuman EM. Labeling, detection and identification of newly synthesized proteomes with bioorthogonal non-canonical amino-acid tagging. Nat Protoc. 2 England 2007 p. 532–40. 17406607 10.1038/nprot.2007.52

[12] Cui SJ, Xu LL, Zhang T, Xu M, Yao J, Fang CY, et al Proteomic characterization of larval and adult developmental stages in Echinococcus granulosus reveals novel insight into host-parasite interactions. J Proteomics. 2013 10.1016/j.jprot.2013.04.013 . 23603110

[13] Monteiro KM, de Carvalho MO, Zaha A, Ferreira HB. Proteomic analysis of the Echinococcus granulosus metacestode during infection of its intermediate host. Proteomics. 2010;10(10):1985–99. 10.1002/pmic.200900506 . 20217864

[14] Parkinson J, Wasmuth JD, Salinas G, Bizarro CV, Sanford C, Berriman M, et al A transcriptomic analysis of Echinococcus granulosus larval stages: implications for parasite biology and host adaptation. PLoS Negl Trop Dis. 2012;6(11):e1897 10.1371/journal.pntd.0001897 23209850 PMC3510090

[15] Wei L, Yu Y, Shen Y, Wang MC, Min W. Vibrational imaging of newly synthesized proteins in live cells by stimulated Raman scattering microscopy. Proc Natl Acad Sci U S A. 2013;110(28):11226–31. 10.1073/pnas.1303768110 23798434 PMC3710790

[16] Dieterich DC, Link AJ, Graumann J, Tirrell DA, Schuman EM. Selective identification of newly synthesized proteins in mammalian cells using bioorthogonal noncanonical amino acid tagging (BONCAT). Proc Natl Acad Sci U S A. 103 United States2006 p. 9482–7. 16769897 10.1073/pnas.0601637103 PMC1480433

[17] Dieterich DC, Hodas JJ, Gouzer G, Shadrin IY, Ngo JT, Triller A, et al In situ visualization and dynamics of newly synthesized proteins in rat hippocampal neurons. Nat Neurosci. 13 United States2010 p. 897–905. 10.1038/nn.2580 20543841 PMC2920597

[18] Kramer G, Sprenger RR, Back J, Dekker HL, Nessen MA, van Maarseveen JH, et al Identification and quantitation of newly synthesized proteins in Escherichia coli by enrichment of azidohomoalanine-labeled peptides with diagonal chromatography. Mol Cell Proteomics. 2009;8(7):1599–611. 10.1074/mcp.M800392-MCP200 19321432 PMC2709250

[19] Hinz FI, Dieterich DC, Tirrell DA, Schuman EM. Non-canonical amino acid labeling in vivo to visualize and affinity purify newly synthesized proteins in larval zebrafish. ACS Chem Neurosci. 2012;3(1):40–9. 10.1021/cn2000876 22347535 PMC3278164

[20] Hinz F, Dieterich D, Schuman E. Teaching old NCATs new tricks: using non-canonical amino acid tagging to study neuronal plasticity. Curr Opin Chem Biol. 2013 10.1016/j.cbpa.2013.07.021 . 23938204 PMC5321483

[21] Zhang J, Ye B, Kong J, Cai H, Zhao Y, Han X, et al In vitro protoscolicidal effects of high-intensity focused ultrasound enhanced by a superabsorbent polymer. Parasitol Res. 2013;112(1):385–91. 10.1007/s00436-012-3176-3 . 23086446

[22] Balbinotti H, Santos GB, Badaraco J, Arend AC, Graichen D, Haag KL, et al Echinococcus ortleppi (G5) and Echinococcus granulosus sensu stricto (G1) loads in cattle from Southern Brazil. Vet Parasitol. 2012;188(3–4):255–60. 10.1016/j.vetpar.2012.04.007 . 22571833

[23] Smyth J. Cestoda In: Smyth J, editor. In vitro Cultivation of Parasitic Helminthes. London: CRC press; 1990 p. 123–37.

[24] Chambers MC, Maclean B, Burke R, Amodei D, Ruderman DL, Neumann S, et al A cross-platform toolkit for mass spectrometry and proteomics. Nat Biotechnol. 2012;30(10):918–20. 10.1038/nbt.2377 23051804 PMC3471674

[25] Tsai IJ, Zarowiecki M, Holroyd N, Garciarrubio A, Sanchez-Flores A, Brooks KL, et al The genomes of four tapeworm species reveal adaptations to parasitism. Nature. 2013;496(7443):57–63. 10.1038/nature12031 . 23485966 PMC3964345

[26] Keller A, Nesvizhskii AI, Kolker E, Aebersold R. Empirical statistical model to estimate the accuracy of peptide identifications made by MS/MS and database search. Anal Chem. 2002;74(20):5383–92. . 12403597 10.1021/ac025747h

[27] Nesvizhskii AI, Keller A, Kolker E, Aebersold R. A statistical model for identifying proteins by tandem mass spectrometry. Anal Chem. 2003;75(17):4646–58. . 14632076 10.1021/ac0341261

[28] Zybailov B, Mosley AL, Sardiu ME, Coleman MK, Florens L, Washburn MP. Statistical analysis of membrane proteome expression changes in Saccharomyces cerevisiae. J Proteome Res. 2006;5(9):2339–47. 10.1021/pr060161n . 16944946

[29] Cox J, Mann M. MaxQuant enables high peptide identification rates, individualized p.p.b.-range mass accuracies and proteome-wide protein quantification. Nat Biotechnol. 2008;26(12):1367–72. 10.1038/nbt.1511 . 19029910

[30] Powell S, Forslund K, Szklarczyk D, Trachana K, Roth A, Huerta-Cepas J, et al eggNOG v4.0: nested orthology inference across 3686 organisms. Nucleic Acids Res. 2014;42(Database issue):D231–9. 10.1093/nar/gkt1253 24297252 PMC3964997

[31] Banerjee PS, Ostapchuk P, Hearing P, Carrico IS. Unnatural amino acid incorporation onto adenoviral (Ad) coat proteins facilitates chemoselective modification and retargeting of Ad type 5 vectors. J Virol. 2011;85(15):7546–54. 10.1128/JVI.00118-11 21613404 PMC3147895

[32] Hess E. Ultrastructural study of the tetrathyridium of Mesocestoides corti Hoeppli, 1925: tegument and parenchyma. Z Parasitenkd. 1980;61(2):135–59. . 7376694 10.1007/BF00925460

[33] Martínez C, Paredes R, Stock RP, Saralegui A, Andreu M, Cabezón C, et al Cellular organization and appearance of differentiated structures in developing stages of the parasitic platyhelminth Echinococcus granulosus. J Cell Biochem. 2005;94(2):327–35. 10.1002/jcb.20294 . 15526286

[34] Barrett J. Forty years of helminth biochemistry. Parasitology. 2009;136(12):1633–42. 10.1017/S003118200900568X . 19265562

[35] McManus DP. Reflections on the biochemistry of Echinococcus: past, present and future. Parasitology. 2009;136(12):1643–52. 10.1017/S0031182009005666 . 19250598

[36] Constantine CC, Bennet-Jenkins EM, Lymbery AJ, Jenkins DJ, Behm CA, Thompson RC. Factors influencing the development and carbohydrate metabolism of Echinococcus granulosus in dogs. J Parasitol. 1998;84(5):873–81. . 9794623

[37] Kita K, Hirawake H, Takamiya S. Cytochromes in the respiratory chain of helminth mitochondria. Int J Parasitol. 1997;27(6):617–30. . 9229245 10.1016/s0020-7519(97)00016-7

[38] Millerioux Y, Ebikeme C, Biran M, Morand P, Bouyssou G, Vincent IM, et al The threonine degradation pathway of the Trypanosoma brucei procyclic form: the main carbon source for lipid biosynthesis is under metabolic control. Mol Microbiol. 2013;90(1):114–29. 10.1111/mmi.12351 23899193 PMC4034587

[39] Folk P, Půta F, Skruzný M. Transcriptional coregulator SNW/SKIP: the concealed tie of dissimilar pathways. Cell Mol Life Sci. 2004;61(6):629–40. 10.1007/s00018-003-3215-4 . 15052407 PMC11138892

[40] Bai Y, Zhang Z, Jin L, Kang H, Zhu Y, Zhang L, et al Genome-wide sequencing of small RNAs reveals a tissue-specific loss of conserved microRNA families in Echinococcus granulosus. BMC Genomics. 2014;15:736 10.1186/1471-2164-15-736 25168356 PMC4156656

[41] Woodman PG, Futter CE. Multivesicular bodies: co-ordinated progression to maturity. Curr Opin Cell Biol. 2008;20(4):408–14. 10.1016/j.ceb.2008.04.001 18502633 PMC2577128

[42] Wollert T, Hurley JH. Molecular mechanism of multivesicular body biogenesis by ESCRT complexes. Nature. 2010;464(7290):864–9. 10.1038/nature08849 20305637 PMC2851844

[43] Wang H, Liu J, Wang F, Chen M, Xiao Z, Ouyang R, et al The role of charged multivesicular body protein 5 in programmed cell death in leukemic cells. Acta Biochim Biophys Sin (Shanghai). 2013;45(5):383–90. 10.1093/abbs/gmt028 . 23619569

[44] McMahon HT, Mills IG. COP and clathrin-coated vesicle budding: different pathways, common approaches. Curr Opin Cell Biol. 2004;16(4):379–91. 10.1016/j.ceb.2004.06.009 . 15261670

[45] Majeed SR, Vasudevan L, Chen CY, Luo Y, Torres JA, Evans TM, et al Clathrin light chains are required for the gyrating-clathrin recycling pathway and thereby promote cell migration. Nat Commun. 2014;5:3891 10.1038/ncomms4891 24852344 PMC4050264

[46] Brodsky FM. Diversity of clathrin function: new tricks for an old protein. Annu Rev Cell Dev Biol. 2012;28:309–36. 10.1146/annurev-cellbio-101011-155716 . 22831640

[47] Buck AH, Coakley G, Simbari F, McSorley HJ, Quintana JF, Le Bihan T, et al Exosomes secreted by nematode parasites transfer small RNAs to mammalian cells and modulate innate immunity. Nat Commun. 2014;5:5488 10.1038/ncomms6488 25421927 PMC4263141

[48] Twu O, Johnson PJ. Parasite extracellular vesicles: mediators of intercellular communication. PLoS Pathog. 2014;10(8):e1004289 10.1371/journal.ppat.1004289 25166021 PMC4148435

[49] Regev-Rudzki N, Wilson DW, Carvalho TG, Sisquella X, Coleman BM, Rug M, et al Cell-cell communication between malaria-infected red blood cells via exosome-like vesicles. Cell. 2013;153(5):1120–33. 10.1016/j.cell.2013.04.029 . 23683579

[50] Lightowlers MW. Vaccination against hydatid disease. Dev Biol (Basel). 2002;110:81–7. . 12477310

