# Supplementary figures and images for: Identification of Newly Synthesized Proteins by Echinococcus granulosus Protoscoleces upon Induction of Strobilation
Source: PLoS Negl Trop Dis. 2015 Sep 22;9(9):e0004085. doi: 10.1371/journal.pntd.0004085 (PMC4578768; doi:10.1371/journal.pntd.0004085)

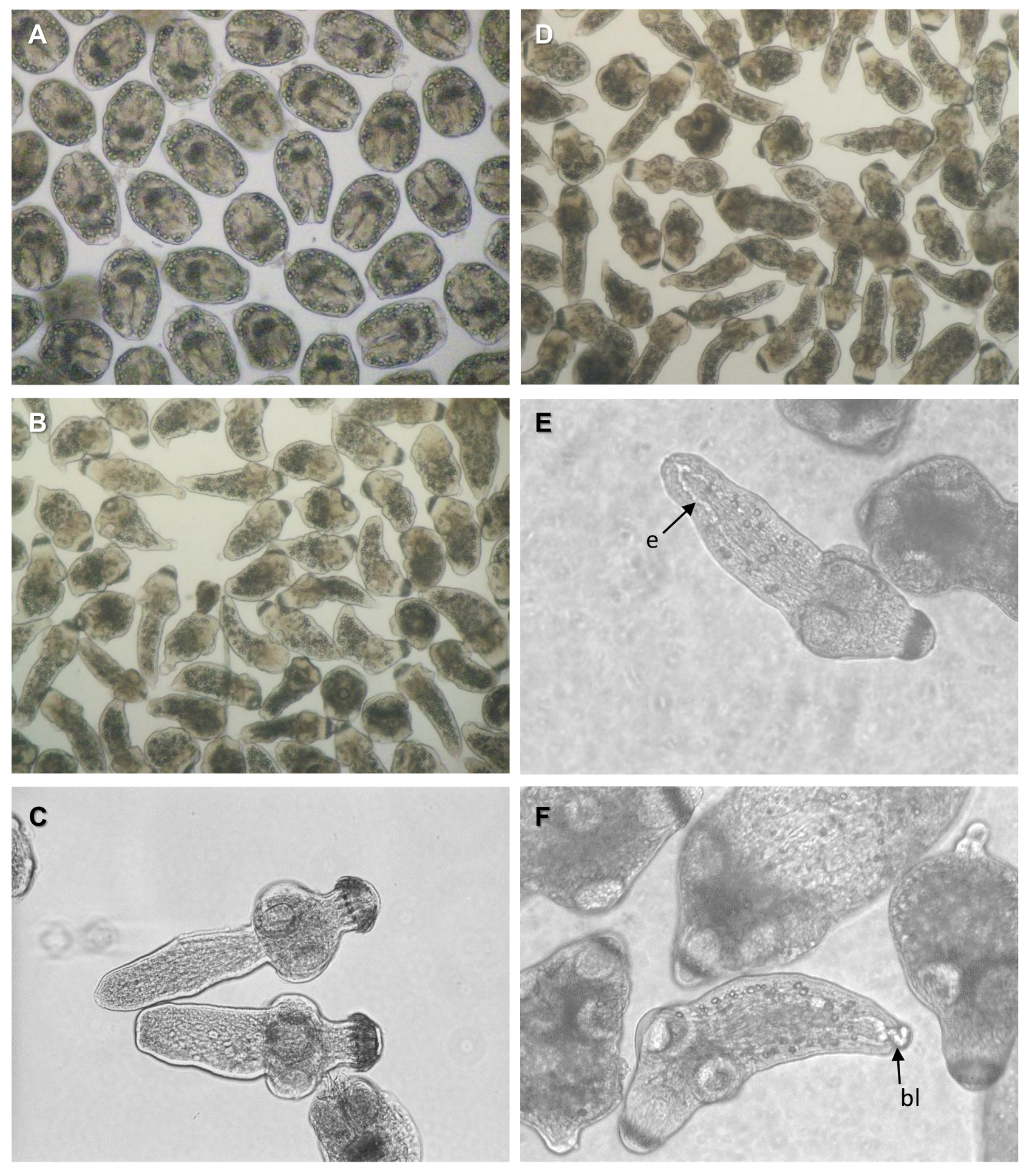

Supplement: S1 Fig — PSCs after pepsin treatment (A) and after 3 days in culture without stimuli for strobilar development (B). After one week, NSD worms present a small number of calcareous corpuscles (C). PCSs after 3 days in complete biphasic medium (D). After 5 days, calcareous corpuscles are much reduced and excretory canals (e) become evident (E) and, after one week, posterior excretory bladder (bl) is also visible (F) Magnification, 100x (A, B and D) and 200x (C, E and F). (TIF) [file pntd.0004085.s001.tif]

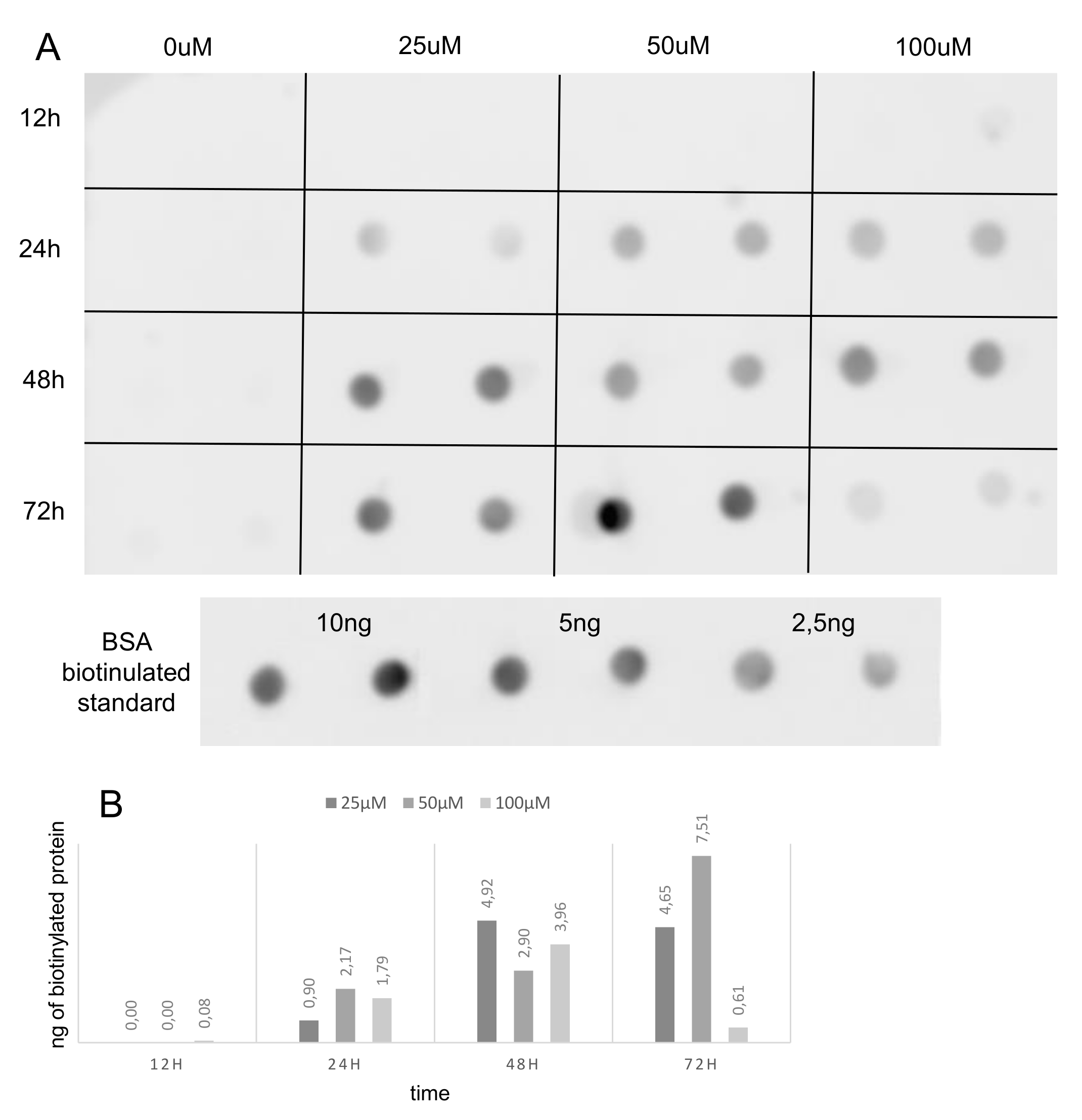

Supplement: S2 Fig — After incubation with 0–100 μM AHA for 12−72 h, the biotin-labeled AHA-containing NSPs were detected by immunoblot (A) and quantified (B) by comparing the intensities of the sample dots and the biotinylated Bovine Serum Albumin standard dots. (TIF) [file pntd.0004085.s002.tif]
