# Supplementary material for: Identification of Newly Synthesized Proteins by Echinococcus granulosus Protoscoleces upon Induction of Strobilation
Source: PLoS Negl Trop Dis. 2015 Sep 22;9(9):e0004085. doi: 10.1371/journal.pntd.0004085 (PMC4578768; doi:10.1371/journal.pntd.0004085)
Supplement: S1 Text — Additional methods and results used in genomic data analysis, PCS cultivation and dot blot assays. (DOCX) [file pntd.0004085.s004.docx]

**Supplemental information**

**Genomic data analysis**

The genomic data for *E. granulosus* were analyzed with a Python script to confirm the feasibility of AHA labeling because only proteins that contain at least one non-terminal methionine residue can be labeled. There are 10,445 annotated proteins in the genomic database available from GeneDB [1]. Of these, 94.5% have Met residues other than the initial Met. There were 11,319 protein sequences predicted from the CHGCS genomic database [2], with 94.9% of them containing Met residues other than the N-terminal Met. From the GeneDB database, we also quantified the percentage of predicted proteins that contained Met residues other than the N-terminal Met for *Echinococcus* *multilocularis* (95.5%), *Schistosoma* *mansoni* (94.1%) and *Taenia* *solium* (94.4%). These findings are comparable to that of mammals, with at least 94% [3], but just a little lower than that of *Danio rerio* (97.97%) [4].

**PSCs cultivation**

PSCs were incubated in different conditions which could stimulate the development of the strobilar stage of *E. granulosus* and enable AHA incorporation. The modifications introduced in the protocol previously described by Smyth [5], apparently did not change the strobilation induction. In this way, even after decreasing the concentration of fetal bovine serum and removal of yeast extract (both potential source of Met) and Met from the strobilation medium, it remained a satisfactory trigger for morphological changes. Calcareous corpuscles became less dense (48h) and excretory canals (120h) and posterior excretory bladder (168h) became clear, both changes being consistent with the described by Smyth strobilar development (S1 Fig). In each experiment, an aliquot was maintained in culture to confirm these morphological alterations. No changes were observed in the PSCs viability in the presence of AHA, thus demonstrating the non-toxicity of AHA to *E. granulosus* PSCs.

**Dot blot assay**

For dot blot (S2 Fig), we used the Click-iT Biotin Protein Analysis Detection Kit (Invitrogen) to label AHA-containing NSPs with biotin alkyne. 10 µg of biotin-labeled proteins were manually applied to a PVDF membrane (Hybond-P, GE Healthcare) following the manufactures’ instructions. The biotin-labeled AHA-containing NSPs were then detected using streptavidin-horseradish peroxidase (diluted 1:10,000 v/v) and ECL detection reagent (ECL protein biotinylation module, GE Healthcare) and imaged in a VersaDoc imaging system (Bio-Rad). The amount of biotinylated NSP was estimated with the software ImageJ by comparing the intensity of the sample dots with the biotinylated Bovine Serum Albumin (Sigma) standard dots.

1. Tsai IJ, Zarowiecki M, Holroyd N, Garciarrubio A, Sanchez-Flores A, Brooks KL, et al. The genomes of four tapeworm species reveal adaptations to parasitism. Nature. 2013;496(7443):57-63. doi: 10.1038/nature12031. PubMed PMID: 23485966.

2. Zheng H, Zhang W, Zhang L, Zhang Z, Li J, Lu G, et al. The genome of the hydatid tapeworm Echinococcus granulosus. Nat Genet. 2013. doi: 10.1038/ng.2757. PubMed PMID: 24013640.

3. Dieterich DC, Lee JJ, Link AJ, Graumann J, Tirrell DA, Schuman EM. Labeling, detection and identification of newly synthesized proteomes with bioorthogonal non-canonical amino-acid tagging. Nat Protoc. 2. England2007. p. 532-40.

4. Hinz FI, Dieterich DC, Tirrell DA, Schuman EM. Non-canonical amino acid labeling in vivo to visualize and affinity purify newly synthesized proteins in larval zebrafish. ACS Chem Neurosci. 2012;3(1):40-9. doi: 10.1021/cn2000876. PubMed PMID: 22347535; PubMed Central PMCID: PMCPMC3278164.

5. Smyth J. Cestoda. In: Smyth J, editor. In vitro Cultivation of Parasitic Helminthes. London: CRC press; 1990. p. 123-37.
